# Association between endothelial function and skin advanced glycation end-products (AGEs) accumulation in a sample of predominantly young and healthy adults

**DOI:** 10.1186/s12933-024-02428-3

**Published:** 2024-09-09

**Authors:** Juanita J. Fewkes, Aimee L. Dordevic, Margaret Murray, Gary Williamson, Nicole J. Kellow

**Affiliations:** 1 Department of Nutrition, Dietetics and Food, Faculty of Medicine, Nursing and Health Sciences, Monash University, 264 Ferntree Gully Road, Notting Hill, 3168 Australia; 2 Victorian Heart Institute, Victoria Heart Hospital, 631 Blackburn Road, Clayton, VIC 3168 Australia; 3 School of Chemistry, Faculty of Science, Monash University, Clayton, VIC 3800 Australia; 4 Centre for Innate Immunity and Infectious Diseases, Hudson Institute of Medical Research, Clayton, VIC Australia

**Keywords:** Advanced glycation end products, AGEs, Cardiovascular risk, Endothelial function, Endothelium-dependent vasodilation, Flow-mediated dilation, Skin autofluorescence, Vascular endothelium

## Abstract

**Background:**

In populations with chronic disease, skin autofluorescence (SAF), a measure of long-term fluorescent advanced glycation end-products (AGEs) accumulation in body tissues, has been associated with vascular endothelial function, measured using flow-mediated dilation (FMD). The primary aim of this study was to quantify the relationship between endothelial function and tissue accumulation of AGEs in adults from the general population to determine whether SAF could be used as a marker to predict early impairment of the endothelium.

**Methods:**

A cross-sectional study was conducted with 125 participants (median age: 28.5 y, IQR: 24.4–36.0; 54% women). Endothelial function was measured by fasting FMD. Skin AGEs were measured as SAF using an AGE Reader. Participant anthropometry, blood pressure, and blood biomarkers were also measured. Associations were evaluated using multivariable regression analysis and were adjusted for significant covariates.

**Results:**

FMD was inversely correlated with SAF (ρ = -0.50, *P* < 0.001) and chronological age (ρ = -0.51, *P* < 0.001). In the multivariable analysis, SAF, chronological age, and male sex were independently associated with reduced FMD (B [95% CI]; -2.60 [-4.40, -0.80]; -0.10 [-0.16, -0.03]; 1.40 [0.14, 2.67], respectively), with the multivariable model adjusted R^2^ = 0.31, *P* < 0.001.

**Conclusions:**

Higher skin AGE levels, as measured by SAF, were associated with lower FMD values, in a predominantly young, healthy population. Additionally, older age and male participants exhibited significantly lower FMD values, corresponding with compromised endothelial function. These results suggest that SAF, a simple and inexpensive marker, could be used to predict endothelial impairment before the emergence of any structural artery pathophysiology or classic cardiovascular disease risk markers.

**Trial registration:**

The study was prospectively registered with the Australian New Zealand Clinical Trials Registry (ACTRN12621000821897) and concurrently entered into the WHO International Clinical Trials Registry Platform under the same ID number.

**Supplementary Information:**

The online version contains supplementary material available at 10.1186/s12933-024-02428-3.

## Background

Endothelial dysfunction is a predictive risk factor for the development of atherosclerosis and cardiovascular disease (CVD), the leading cause of death worldwide [1]. Atherosclerosis, a chronic inflammatory disease, is the slow progressive accumulation of fatty plaque in the artery wall, causing narrowing and impairment of blood flow [2]. The subclinical asymptomatic changes that occur in the artery can be assessed by specific early-stage tests, including endothelial function by flow-mediated dilation (FMD), arterial stiffness via pulse-wave velocity (PWV), and artery structure through intima-media thickness (IMT) of the carotid artery.

Advanced glycation end-products (AGEs) are a diverse group of compounds formed by the irreversible reaction between amino acids and reducing sugars [3]. AGEs are formed endogenously and accumulate in body tissues throughout life [4–6] and are also derived exogenously from the diet [7]. The accumulation of AGEs in tissues is elevated in individuals with metabolic dysfunction [8], type 1 diabetes [9], type 2 diabetes [10], and renal disease [11]. AGEs can adversely affect the vasculature via pro-atherogenic mechanistic pathways, increasing endothelial dysfunction. AGEs can form cross-links between long-lived extracellular matrix (ECM) proteins such as elastin and collagen [12], which leads to increased artery stiffness [13]. AGEs can also bind with the receptor for AGEs (RAGE) to contribute to downstream proinflammatory signaling, including leading to an increase in oxidative stress by reducing nitric oxide (NO) or endothelial nitric oxide synthase (eNOS) expression, as shown through mechanistic studies [14–19]. RAGE is expressed in many cell types, including vascular endothelial cells [20].

Circulating AGEs concentrations are transient and do not necessarily directly reflect the chronic accumulation of AGEs in tissues [21]. In contrast, long-term deposition of AGEs in tissue can be non-invasively estimated by measuring skin autofluorescence (SAF) using a desktop device known as an AGE Reader [4]. AGEs accumulation in skin tissue measured by the AGE Reader positively correlates with PWV and carotid IMT markers in populations predominantly with chronic disease [22]. Furthermore, there is a positive association between SAF and vascular stiffness, measured by PWV, where the relationship is most marked in men and younger individuals, regardless of glycemic status [23]. While PWV and carotid IMT are structural atherosclerotic biomarkers, FMD is a vascular functional biomarker. Thus, FMD can be used to assess nitric oxide-mediated endothelial dysfunction before any structural changes in the artery occur, providing early prognostic information about endothelial function [24]. Inverse correlations have previously been reported between tissue AGEs accumulation and FMD in four at-risk populations: subjects with uremia [25], older subjects with moderate-to-high CVD risk factors but without chronic kidney disease [25], subjects with diabetes mellitus [26], and community-dwelling older women [27]. As FMD is a functional prognostic marker, tissue AGEs accumulation, measured as SAF, could play an important early role in endothelial dysfunction in apparently healthy people before they develop any classic CVD risk factors such as elevated waist circumference, blood pressure, and LDL cholesterol.

The primary aim of this study was to quantify the relationship between endothelial function, via FMD, and tissue accumulation of AGEs, via SAF, in adults from the general population, to determine whether SAF could be used as a marker to predict early impairment of the endothelium. The secondary aim was to identify clinical risk factors for CVD that could influence the relationship between SAF and FMD.

## Methods

### Study design and participants

**Fig. 1:**
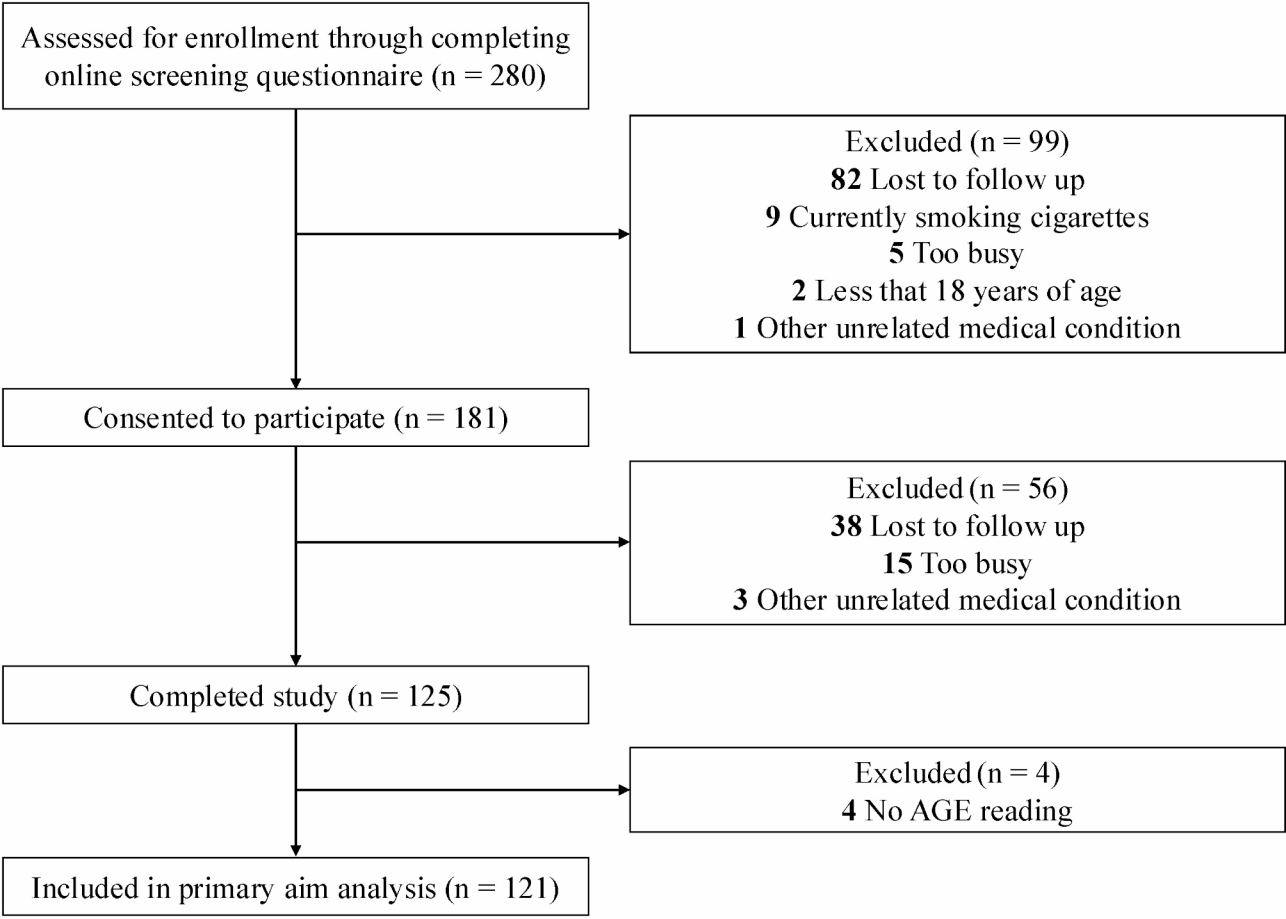
Flow diagram of participant inclusion. AGE, advanced glycation end-products

A cross-sectional study on a general population-based cohort was conducted between 2021 and 2022 in Melbourne, Australia. The exclusion criteria were: (1) age < 18 years, (2) current smokers or those who had ceased smoking within six months of testing, (3) women who were pregnant or breastfeeding, or (4) individuals who had an arteriovenous fistula, implantable cardiac defibrillator, cardiac pacemaker, or had made any dramatic dietary changes in the previous three months prior to testing. The study was conducted in accordance with the Declaration of Helsinki for human studies and was approved by the Monash University Human Research Ethics Committee (Project ID: 23731). The study was prospectively registered with the Australian New Zealand Clinical Trials Registry (ACTRN12621000821897). An online screening questionnaire via Qualtrics (Qualtrics, Provo, Utah, USA) was utilized to confirm eligibility before study enrolment. Informed consent was obtained from all participants prior to screening and enrolment. Once enrolled, participants completed a general medical, demographic, physical activity status, and lifestyle online questionnaire, then were invited for a testing visit in the morning following an overnight fast (≥ 12 h). Participants were instructed to avoid exercise for 24 h prior to testing. All medications being taken by participants were noted, along with female participants’ menstrual phase and cycle length. The flow diagram of participant inclusion is displayed in Fig. 1.

### Endothelial function

A trained examiner assessed brachial artery endothelial function via FMD, using a 7–12 MHz multifrequency linear array probe attached to a high-resolution ultrasound machine (Venue Go, GE Healthcare, Massachusetts, United States) according to recent expert guidelines [24]. In brief, after supine rest in a semi-darkened, temperature-controlled room, the participant’s left arm was extended from the torso, and a rapid inflation pneumatic cuff (SC12D, Hokanson, Bellevue, WA) was wrapped immediately below the elbow. The brachial artery was scanned in B-mode 2–6 cm longitudinally above the antecubital fossa in the distal third of the upper arm. After an optimal image of the artery diameter was obtained, the probe was held in place until the conclusion of the protocol. Baseline artery diameter was continuously recorded for 1 min, followed by 15 s of recorded pulsed-wave Doppler flow. The pneumatic cuff was then rapidly inflated on the forearm to 220 mmHg for 5 min to produce lower forearm ischemia and induce shear stress. Thirty seconds before cuff deflation, the continuous recording was resumed. Live acquisition of pulsed-wave Doppler flow mode occurred for the first 15 s post-cuff deflation [28] before switching back to B-mode for artery diameter assessment. Recording continued for 3 min post-cuff deflation. The recorded files were analyzed by a trained blinded observer using an offline continuous automated edge-detection software Brachial Analyzer (Medical Imaging Applications, LLC, Iowa, USA) [29]. FMD was calculated as the percentage change in peak reactive hyperemia diameter from baseline artery diameter. The mean shear rate area under the curve was calculated for the 15 s of Doppler flow, and the calculation for shear rate was defined as shear rate = 8 x (mean blood flow velocity [cm/s] / diameter [cm]) [24]. As initial baseline artery diameter can influence the assessment of the percentage change in FMD, all analyses were also conducted on allometrically scaled FMD data according to the method described by Atkinson and Batterham [30].

### Skin autofluorescence

Skin autofluorescence (SAF), a marker of long-term AGE accumulation in the skin, was measured using the AGE Reader (Diagnoptics BV, Groningen, The Netherlands). Certain AGEs display fluorescent properties, allowing the AGE Reader to estimate the level of AGE accumulation through excitation via UV light, which penetrates the dermis (methods have been described elsewhere in detail) [4, 31]. Measurements were taken on the ventral surface of the lower forearm, with researchers choosing the lightest section of skin, an approximate 1 cm^2^ area, free from dark marks or skin abnormalities. Per the manufacturer’s instructions, SAF was measured in triplicate, averaged, and expressed in arbitrary units (AU). The AGE Reader has an intra-individual overall Altman error percentage of 5.03% over repeated SAF measurements on a single day and a 5.87% error percentage for seasonal changes [4].

### Anthropometric measures and physical activity

Height was measured twice and averaged using a stadiometer to the nearest 1 mm. Waist circumference was measured twice and averaged to the nearest 1 mm using a constant tension flexible tape measure at the mid-point between the inferior margin of the last rib and the iliac crest [32]. Body mass index (BMI) was calculated as weight (kg) divided by height (m) squared. Weight and body composition were measured using a bioelectrical impedance analysis scale (SECA, 515, Ecomed). Variables, including total body fat-free mass (kg and %), total body fat mass (kg and %), total body water (L and %), total skeletal muscle mass (kg and %), and visceral adipose tissue (L and %) were recorded. The International Physical Activity Questionnaire (IPAQ)- short form was self-administered to assess participants’ habitual physical activity levels [33].

### Blood pressure

After 10 min of supine rest in a quiet room, brachial systolic, diastolic, and mean arterial blood pressure were measured with an automated oscillometric blood pressure monitor (Welch Allyn ProBP 3400 Digital). The average of three consecutive congruent measurements defined blood pressure and heart rate.

### Biochemical parameters

Fasting plasma samples assessed glucose, insulin, and soluble receptor for advanced glycation end-products (sRAGE). Fasting serum samples assessed total cholesterol, high-density lipoprotein (HDL) cholesterol, low-density lipoprotein (LDL) cholesterol, triglycerides, and high-sensitivity C-reactive protein (hsCRP). Whole blood was collected for the assessment of glycated hemoglobin (HbA_1c_). All fasting blood samples were processed and stored at -80 °C.

Plasma glucose, serum lipids, and hsCRP analysis were performed on an Indiko Clinical Chemistry Analyzer (Thermo Fisher Scientific Oy, Vantaa, Finland) using commercially available kits from the manufacturer. Plasma insulin was measured by a human insulin ELISA kit (EZHI-14 K, Merck Millipore, Massachusetts, USA), following the manufacturer’s instructions. Plasma sRAGE was determined through the Quantikine ELISA Human RAGE Immunoassay kit (R&D Systems, Minnesota, USA), following the manufacturer’s protocols. Both sRAGE and insulin were read on an absorbance plate reader (PHERAstar FS, BMG Labtech, Ortenberg, Germany). HbA_1c_ was analyzed using the Capillary 3 HbA_1c_ kit (Sebia, Lisses, France) by Monash Pathology (Monash Medical Centre, Clayton). Homeostatic model assessment for insulin resistance (HOMA-IR) was calculated with the formula [fasting insulin (µU/mL) × fasting glucose (mmol/L)] ÷ 22.5 [34].

### Sample size calculation

Based on alpha = 0.05, a power of 80%, and an estimated correlation coefficient of 0.25 or greater between FMD and SAF, a minimum of 120 participants were required.

### Statistical analysis

Unless otherwise stated, all data are presented as median (interquartile range) for continuous variables and numbers (percentage) for categorical variables. Participants with missing data were excluded from the analysis. The data were tested for normality using the Kolmogorov-Smirnov test and visual inspection of box plots and histograms using Stata, version 18.0 (StataCorp, College Station, Texas, USA). Correlation analysis was used to quantify the relationship between FMD, SAF, and other clinical variables. Statistically significant variables were then included in a multivariable linear regression model with FMD as the dependent variable. Multivariable regression model 1 was constructed to include sex and age. From the correlation analysis, variables correlated with FMD (*p* ≤ 0.05) were entered into model 2. Furthermore, variables with a known association with CVD were also entered into model 2 to identify independent predictors of FMD. All variables were tested for collinearity via the vif command in Stata. Two-tailed p values ≤ 0.05 were considered statistically significant.

## Results

**Fig. 2:**
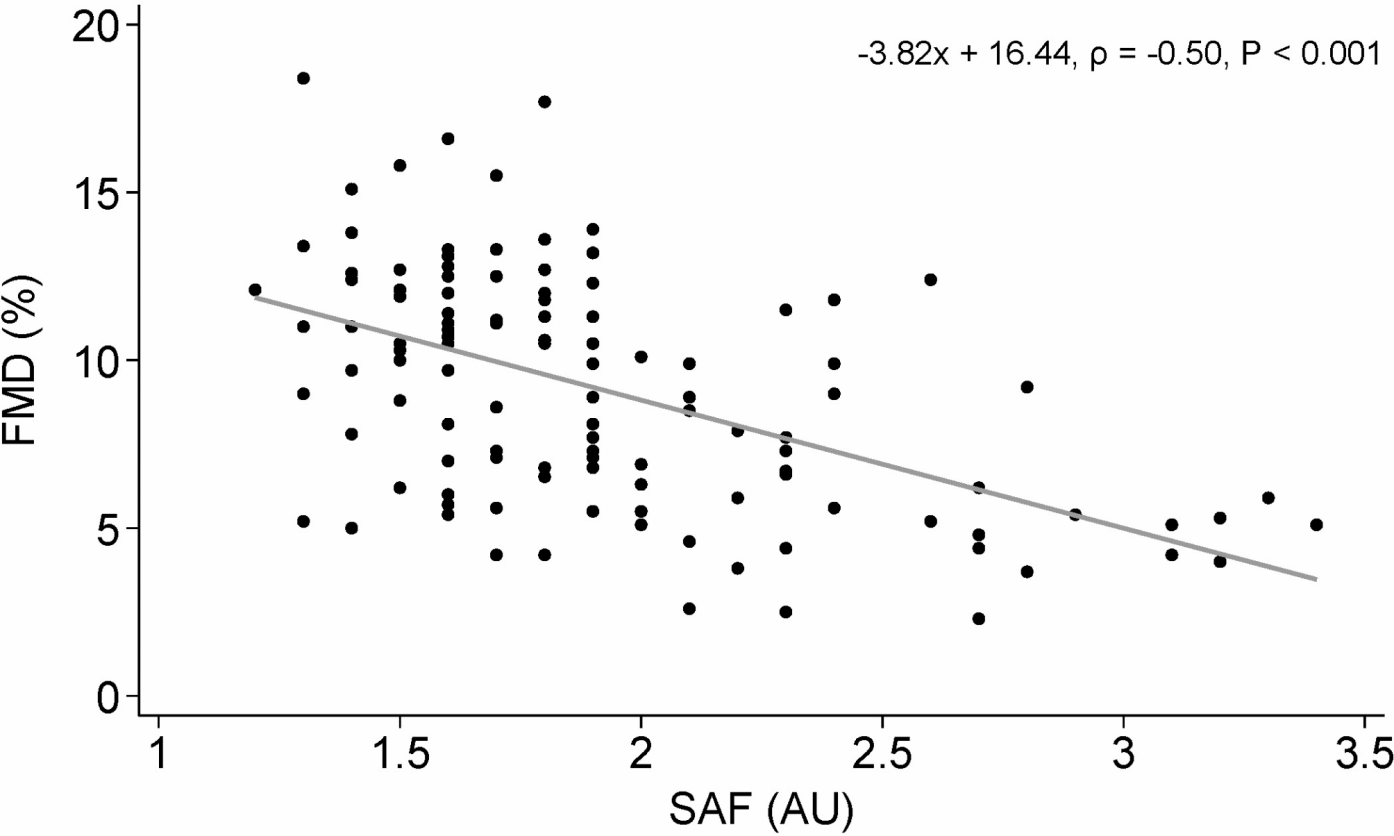
Unadjusted linear regression and Spearman correlation analysis to evaluate the association between SAF and FMD. *N* = 121. Scatter plot with linear trend line (solid line). Abbreviations: SAF, skin autofluorescence; AU, arbitrary units; FMD, flow-mediated dilation; N, sample size

One hundred and twenty-five participants completed a testing visit; baseline characteristics are presented in Table 1, of which data for SAF was missing in 4 individuals. Thus, 121 participants were included in the primary outcome analysis of the relationship between FMD and SAF. While the cohort characteristics were heterogeneous, each variable’s median and interquartile range was generally within the healthy range; thus, the cohort could be considered predominantly young and healthy adults. In unadjusted linear regression and Spearman correlation analysis, SAF was inversely associated with FMD (ρ = -0.50, *P* < 0.001, *N* = 121) (Fig. 2). When participants with medical conditions and those who were aged over 50 y were removed from the sample, SAF remained inversely associated with FMD (ρ = -0.31, *P* < 0.01, *N* = 92).

**Table 1:** Participant characteristics of study subjects

	Median (IQR) or number (%)	Range
Age, years	28.5 (24.4–36.0)	18.7–72.8
Sex, female	68 (54.4%)	
Skin autofluorescence, AU (*N* = 121)	1.8 (1.6–2.1)	1.2–3.4
FMD, %	9.7 (6.0-11.9)	2.3–18.4
Allometrically scaled FMD, %	8.0 (6.9–9.2)	5.6–12.4
Baseline diameter, mm	3.6 (3.1–4.1)	2.4-5.0
Peak diameter, mm	4.0 (3.4–4.4)	2.7–5.4
Absolute FMD change, mm	0.3 (0.2–0.4)	0.1–0.7
Shear rate, sec^−1^	1415.1 (1039.4-1743.7)	712.7-2545.5
BMI, kg/m^2^	22.9 (20.3–25.7)	16.1–38.2
Waist circumference, cm	80.0 (72.5–90.0)	57.8–127.0
Fat-free mass, %	72.7 (68.9–80.0)	51.8–94.1
Fat mass, %	27.3 (20.0-31.1)	5.9–48.2
Total body water, %	53.3 (50.0-58.3)	38.2–67.3
Skeletal muscle mass, % (*N* = 102)	34.9 (31.2–38.3)	20.6–45.4
Visceral adipose tissue, %	2.2 (1.5–2.9)	0.1–7.1
Physical activity, MET minutes/week	2399 (1138–4320)	0-10479
Systolic blood pressure, mmHg	108.3 (101.7–116.0)	90.3-155.3
Diastolic blood pressure, mmHg	68.7 (64.0–73.0)	56.3–90.7
Mean arterial pressure, mmHg	81.6 (76.4–87.7)	67.7-108.7
Heart rate, beats/min	60.0 (52.3–67.3)	37.0-90.7
Glucose, mmol/L (*N* = 117)	5.3 (5.0-5.5)	4.4–6.8
Insulin, pmol/ml (*N* = 117)	32.5 (21.3–44.0)	5.1-170.4
sRAGE, pg/mL (*N* = 117)	1832 (1296–2384)	363–4731
Total Cholesterol, mmol/L (*N* = 117)	5.0 (4.4–5.9)	3.0-22.2
LDL Cholesterol, mmol/L (*N* = 117)	3.2 (2.5-4.0)	1.4–20.2
HDL Cholesterol, mmol/L (*N* = 117)	1.7 (1.5-2.0)	0.9–3.2
Triglycerides, mmol/L (*N* = 117)	0.8 (0.7–1.2)	0.3–3.5
hsCRP, mg/l (*N* = 117)	0.6 (0.3–1.4)	0.0-10.7
HbA_1c_, % (*N* = 117)	5.1 (5.0-5.3)	4.4–6.6
HOMA-IR, (*N* = 117)	1.3 (0.9–1.8)	0.2–7.4

*N* = 125 unless otherwise stated. Values are shown as median (IQR) or number (percentage). Abbreviations: AU, arbitrary units; BMI, body mass index; FMD, flow-mediated dilation; HbA_1c_, glycated hemoglobin; HDL, high-density lipoprotein; HOMA-IR, homeostatic model assessment for insulin resistance; hsCRP, high-sensitivity C-reactive protein; IQR, interquartile range; LDL, low-density lipoprotein; MET, metabolic equivalent task; sRAGE, soluble receptor for advanced glycation end-products

### Relationships between FMD, SAF, and other predictor variables

Correlation analysis shows the relationships between FMD and other study variables (Table 2). Variables associated with FMD included chronological age (ρ = -0.51, *P* < 0.001), SAF (ρ = -0.50, *P* < 0.001), visceral adipose tissue (ρ = -0.24, *P* < 0.01), total cholesterol (ρ = -0.21, *P* < 0.05), LDL cholesterol (ρ = -0.23, *P* < 0.01), and HbA1c (ρ = -0.28, *P* < 0.01). There were no statistically significant relationships between FMD and the other covariates.

In multivariable model 1, FMD was negatively associated with chronological age (B = -0.12; 95% CI, -0.16, -0.08; *P* < 0.001) (Table 3). Only significant variables from the correlation analysis, with the removal of collinear variables (total cholesterol) plus the addition of any variables with known biological associations with CVD or FMD (sex, waist circumference, and systolic BP), were included in the multivariable model 2 (Table 3). After the exclusion of 12 participants who had incomplete data, FMD was associated with age (B = -0.10; 95% CI, -0.16, -0.03; *P* = 0.005), sex (B = 1.40; 95% CI, 0.14, 2.67; *P* = 0.030), SAF (B = -2.60; 95% CI, -4.40, -0.80; *P* = 0.005), and systolic blood pressure (B = 0.07; 95% CI, 0.01, 0.14; *P* = 0.033) (*N* = 113). Waist circumference, visceral adipose tissue, LDL cholesterol, and HbA_1c_ were not independently associated with FMD after adjustment in the regression model. The combined covariates in model 2 explained 31% of the variance in FMD.

**Table 2:** Spearman correlations between FMD and other study variables

	FMD	AlloFMD	Age	SAF^^^	BMI	WC	FFM	FM	BW	SMM^#^	VAT	PA	SBP	DBP	MAP	HR	Glu^§^	Insulin^§^	sRAGE^§^	TC^§^	LDL C^§^	HDL C^§^	TG^§^	hsCRP^§^	HbA_1c_^§^	HOMA-IR^§^
FMD																										
AlloFMD	0.93‡																									
Age	-0.51‡	-0.56‡																								
SAF^^^	-0.50‡	-0.45‡	0.52‡																							
BMI	-0.02	-0.14	0.32‡	0.16																						
WC	-0.10	-0.23†	0.45‡	0.18*	0.85‡																					
FFM	-0.02	-0.11	-0.21*	-0.22*	-0.56‡	-0.46‡																				
FM	0.02	0.11	0.21*	0.22*	0.56‡	0.46‡	-1.00‡																			
BW	-0.02	-0.12	-0.17	-0.20*	-0.55‡	-0.45‡	0.99‡	-0.99‡																		
SMM^#^	0.05	-0.14	-0.11	-0.28†	-0.25†	-0.20*	0.92‡	-0.92‡	0.92‡																	
VAT	-0.24†	-0.27†	0.38‡	0.42‡	0.48‡	0.60‡	-0.48‡	0.48‡	-0.50‡	-0.44‡																
PA	-0.10	-0.10	-0.01	0.09	-0.15	-0.13	0.32‡	-0.32‡	0.34‡	0.26†	-0.24†															
SBP	-0.16	-0.27†	0.53‡	0.27†	0.41‡	0.47‡	-0.07	0.07	-0.06	0.14	0.34‡	0.10														
DBP	-0.15	-0.18*	0.44‡	0.34‡	0.41‡	0.40‡	-0.25†	0.25†	-0.25†	-0.08	0.39‡	0.03	0.88‡													
MAP	-0.15	-0.22*	0.49‡	0.32‡	0.41‡	0.43‡	-0.17	0.17	-0.17	0.02	0.38‡	0.06	0.96‡	0.98‡												
HR	0.13	0.23†	-0.29†	-0.02	-0.11	-0.12	-0.20*	0.20*	-0.22*	-0.27†	0.05	-0.13	-0.11	0.14	0.03											
Glu^§^	-0.13	-0.20*	0.25†	0.27†	0.36‡	0.38‡	-0.08	0.08	-0.07	-0.01	0.27†	0.10	0.16	0.09	0.12	-0.10										
Insulin^§^	0.16	0.18	-0.05	0.08	0.44‡	0.44‡	-0.50‡	0.50‡	-0.51‡	-0.36‡	0.38‡	-0.14	0.16	0.25†	0.21*	0.25†	0.23†									
sRAGE^§^	-0.01	-0.07	-0.08	-0.18	-0.17	-0.21*	0.31‡	-0.31‡	0.28†	0.25†	-0.26†	0.01	0.01	-0.09	-0.04	0.04	0.02	-0.06								
TC^§^	-0.21*	-0.23*	0.41‡	0.41‡	0.32‡	0.28†	-0.27†	0.27†	-0.26†	-0.23*	0.33‡	0.06	0.30†	0.30‡	0.30‡	-0.06	0.21*	0.07	-0.13							
LDL C^§^	-0.23†	-0.29†	0.41‡	0.41‡	0.38‡	0.36‡	-0.28†	0.28†	-0.28†	-0.22*	0.40‡	0.04	0.35‡	0.33‡	0.34‡	-0.09	0.22*	0.10	-0.15	0.95‡						
HDL C^§^	-0.04	0.10	-0.004	0.01	-0.35‡	-0.39‡	0.04	-0.04	0.06	-0.13	-0.37‡	0.10	-0.22*	-0.17	-0.20*	-0.001	-0.12	-0.28†	0.08	0.22*	-0.01					
TG^§^	-0.05	-0.11	0.27†	0.26†	0.51‡	0.48‡	-0.33‡	0.33‡	-0.36‡	-0.18	0.60‡	-0.12	0.42‡	0.43‡	0.44‡	0.08	0.27†	0.36‡	-0.17	0.36‡	0.41‡	-0.46‡				
hsCRP^§^	0.02	-0.03	0.18*	0.17	0.33‡	0.31‡	-0.30†	0.30†	-0.31‡	-0.17	0.28†	-0.05	0.18*	0.22*	0.21*	-0.05	0.01	0.22*	0.0003	0.19*	0.18*	-0.09	0.33‡			
HbA_1c_^§^	-0.28†	-0.33‡	0.33‡	0.40‡	0.24†	0.30‡	-0.20*	0.20*	-0.19*	-0.20	0.39‡	0.10	0.12	0.16	0.14	-0.08	0.38‡	0.16	-0.10	0.17	0.21*	-0.07	0.17	0.29†		
HOMA-IR^§^	0.15	0.16	-0.02	0.11	0.47‡	0.47‡	-0.50‡	0.50‡	-0.50‡	-0.35‡	0.41‡	-0.14	0.17	0.25†	0.21*	0.25†	0.32‡	0.99‡	-0.07	0.08	0.12	-0.29†	0.37‡	0.21*	0.19*	

*N* = 125 unless otherwise stated. * *P* ≤ 0.05, † *P* ≤ 0.01, and ‡ *P* ≤ 0.001 for Spearman’s ρ correlation coefficient. ^ *N* = 121, # *N* = 102, § *N* = 117. Abbreviations: AlloFMD, allometrically scaled FMD; BMI, body mass index; BW, body water; DBP, diastolic blood pressure; FFM, fat-free mass; FM, fat mass; FMD, flow-mediated dilation; Glu, glucose; HbA1c, glycated hemoglobin; HDL C, high-density lipoprotein; HOMA-IR, homeostatic model assessment for insulin resistance; HR, heart rate; hsCRP, high-sensitivity C-reactive protein; LDL C, low-density lipoprotein; MAP, mean arterial pressure; N, sample size; PA, physical activity; SAF, skin autofluorescence; SBP, systolic blood pressure; SMM, skeletal muscle mass; sRAGE, soluble receptor for advanced glycation end-products; TC, total cholesterol; TG, Triglycerides; VAT, visceral adipose tissue; WC, waist circumference.

**Table 3:** Multivariable regression analysis exploring the associations between FMD and covariates

Covariate	Unstandardized coefficient		Standardized coefficient	t-value	*P*-value	95% CI Lower, Upper	Obs (*N*)	*R*^2^	Adjusted *R*^2^
	B	Std. error	Beta						
*Model 1*									
Intercept	12.73	0.82	0.00	15.43	< 0.001*	11.10, 14.36	125	0.244	0.23
Age	-0.12	0.02	-0.47	-5.90	< 0.001*	-0.16, -0.08			
Sex	0.79	0.57	0.11	1.39	0.167	-0.34, 1.91			
*Model 2*									
Intercept	2.09	7.02	0.000	0.30	0.766	-11.82, 16.00	113	0.359	0.31
Age	-0.10	0.03	-0.37	-2.88	0.005*	-0.16, -0.03			
Sex	1.40	0.64	0.20	2.21	0.030*	0.14, 2.67			
SAF	-2.60	0.91	-0.35	-2.87	0.005*	-4.40, -0.80			
WC	0.05	0.03	0.18	1.49	0.138	-0.02, 0.12			
VAT	-0.17	0.32	-0.06	-0.52	0.607	-0.81, 0.47			
Systolic BP	0.07	0.03	0.25	2.16	0.033*	0.01, 0.14			
LDL Cholesterol	-0.26	0.17	-0.13	-1.50	0.137	-0.59, 0.08			
HbA_1c_ (%)	0.65	1.12	0.06	0.58	0.564	-1.57, 2.87			

Linear multivariable regression analysis was performed to determine the predictors of FMD. Abbreviations: BP, blood pressure; FMD, flow-mediated dilation; HbA_1c_, hemoglobin A1c; LDL, low-density lipoprotein; n, sample size; Obs, observations; SAF, skin autofluorescence; std error, standard error; VAT, visceral adipose tissue; WC, waist circumference.

### Relationships between allometrically scaled FMD, SAF, and other predictor variables

Analyses were also conducted on allometrically scaled FMD data as baseline artery diameter can potentially influence the calculation of percent change FMD. Correlation analysis showed that allometric FMD was correlated with age (ρ = -0.56, *P* < 0.001), SAF (ρ = -0.45, *P* < 0.001), waist circumference (ρ = -0.23, *P* < 0.01), visceral adipose tissue (ρ = -0.27, *P* < 0.01), systolic blood pressure (ρ = -0.27, *P* < 0.01), diastolic blood pressure (ρ = -0.18, *P* < 0.05), mean arterial pressure (ρ = -0.22, *P* < 0.05), heart rate (ρ = 0.23, *P* < 0.01), glucose (ρ = -0.20, *P* < 0.05), total cholesterol (ρ = -0.23, *P* < 0.05), LDL cholesterol (ρ = -0.29, *P* < 0.01), and HbA1c (ρ = -0.33, *P* < 0.00) (Table 2). No statistically significant relationship existed between allometrically scaled FMD and the other covariates. Waist circumference, systolic blood pressure, diastolic blood pressure, mean arterial pressure, heart rate, and glucose were only correlated in the allometric scaled dataset and not in the raw FMD dataset.

Chronological age (B = -0.05; 95% CI, -0.06, -0.03; *P* < 0.001) and sex (B = -0.04; 95% CI, -0.07, -0.02; *P* < 0.01) were negatively associated with allometrically scaled FMD in multivariable model 1 (Additional file 1: Table S1). In model 2 for 113 participants, allometrically scaled FMD was associated with chronological age (B = -0.04; 95% CI, -0.07, -0.02; *P* = 0.001), sex (B = 1.21; 95% CI, 0.70, 1.72; *P* < 0.001), SAF (B = -0.84; 95% CI, -1.54, -0.14; *P* < 0.05), and systolic blood pressure (B = 0.03; 95% CI: 0.01, 0.06; *P* < 0.05) (Additional file 1: Table S1). After adjustment in the regression model, waist circumference, visceral adipose tissue, systolic BP, diastolic BP, heart rate, glucose, total cholesterol, LDL cholesterol, and HbA1c were not independently associated with allometrically scaled FMD. The combined covariates in model 2 explained 40% of the variance in allometrically scaled FMD.

## Discussion

In this study, SAF, age, and sex were associated with brachial artery FMD in a predominantly young and apparently healthy population. Higher SAF levels were associated with lower FMD values, indicating impaired endothelial function. Furthermore, older individuals and males in the sample were more likely to have lower FMD values.

Correlation analysis in the present study showed an inverse correlation of -0.50 between FMD and SAF, which differs from previously reported correlations in some populations with existing metabolic dysfunction. For example, lower inverse correlations were reported in older adults with moderate-to-high CVD risk factors but without chronic kidney disease (*r* = -0.46, *P* < 0.01) [25], adults with diabetes mellitus (*r* = -0.26, *P* = 0.002) [26], and community-dwelling older women (*r* = -0.37, *P* < 0.05) [27]. However, participants with uremia have demonstrated a higher inverse correlation compared with the present study (*r* = -0.80, *P* < 0.01) [25]. Increased tissue AGEs are observed in people with impaired renal function [35]. Kidney dysfunction impairs the urinary excretion of AGEs [36], but the presence of other underlying comorbidities could also contribute to increased AGE generation in people with chronic kidney disease, affecting FMD.

In the multivariable model, our results showed stronger relationships between SAF and FMD in a predominantly young and healthy adult sample than in populations with chronic disease. We found that for every 1 arbitrary unit (AU) increase in SAF, there was a corresponding 2.60% reduction in FMD. In comparison, Wang et al. [25] reported that in patients with uremia on hemodialysis, every 1 AU increase in SAF was associated with a 1.60% reduction in FMD (mean age ± SD: 59.60 ± 11.63 y, *N* = 119). AGEs are not filtered through a hemodialysis membrane, and patients on dialysis longer would possibly have higher AGE accumulation [25], affecting the association with FMD. In people with CVD risk factors but no kidney disease, every 1 AU increase in SAF has been independently associated with a 1.85% reduction in FMD (mean age 58.72 ± 10.50 y, *N* = 57) [25]. However, over half the CVD risk factor group used either angiotensin-converting enzyme inhibitors (ACEIs), angiotensin II receptor blockers (ARBs), beta-blockers, or statins, which can impact FMD. Statins can upregulate eNOS [37] while ACEIs/ARBs reduce angiotensin II levels and increase bradykinin levels [38], both pathways leading to increased NO production and hence vasodilation and improved FMD. Furthermore, SAF is an independent predictor of FMD in participants with diabetes mellitus, though chronological age and BMI were stronger predictors [26]. In diabetes mellitus, other factors can affect NO and oxidative stress, potentially contributing to the FMD and SAF values. For example, increased insulin resistance, postprandial acute hyperglycemia, or chronic hyperglycemia can stimulate O_2_^−^ (superoxide) production, increasing oxidative stress [39, 40], meaning increased endothelial dysfunction misaligned with SAF values.

While most previous studies have recruited participants considered at high risk for CVD, FMD as a prognostic technique is potentially only related to cardiovascular risk factors in low-risk populations [41]. Indeed, participants with older age or CVD risk factors show larger intra-individual variations in FMD, which impair FMD reproducibility [42]. FMD is approximately 70% nitric oxide-mediated [43], and as the artery stiffens with age or more CVD risk factors are present, such as hypertension, hypercholesterolemia, physical inactivity, and diabetes, there may be alternative limitations on the capacity of the artery to dilate. Thus, measuring the FMD in high-risk groups is not entirely an indication of NO-dependent dilation.

Poor nitric oxide production or bioavailability is the precursor to impaired endothelial function and the first step toward atherosclerosis. Mechanistic studies have shown that a greater accumulation of AGEs may directly influence endothelial function through NO. The interaction of AGEs with their receptor RAGE, particularly on endothelial and smooth muscle cells [20], contributes to increased activation of the vascular nicotinamide adenine dinucleotide phosphate (NADPH) oxidase pathway [44, 45], plus increased production of cytokines, chemokines, adhesion molecules and inflammatory factors including transcription factor nuclear factor kappa B (NF-κB) [46, 47]. These factors lead to the generation of reactive oxygen species, including increased superoxide production [40], upregulation of endothelin-1 [48], impairment of eNOS [16, 49], and ultimately quenching or inhibition of NO [14]. Moreover, vasodilation of the artery can also be impaired via AGEs-induced protein cross-linking, altering of extracellular matrix properties [20], and thickening and remodeling of the arterial wall, which can lead to vascular stiffness [50]. Thus, increased skin AGEs accumulation in the dermis and epidermis of the skin is associated with decreased FMD, indicating decreased NO bioavailability, potentially promoting pro-atherogenic effects. SAF measured via the AGE reader is a potential biomarker for early detection of suboptimal endothelial function before any traditional clinical CVD risk factors are present.

In the present study, for every 1-year increase in chronological age, FMD decreased by 0.1%. Vascular aging mechanisms can impact the arterial wall to promote endothelial dysfunction, affecting eNOS through decreased expression, known as eNOS uncoupling [51, 52]. L-arginine and tetrahydrobiopterin (BH4) are cofactors needed to produce NO, which are reduced with age [53, 54]. Baseline artery diameter can overtly influence the FMD percent change calculation. For example, a cross-sectional study with 5695 male subjects aged 30 to 74 showed that larger baseline artery diameter was positively correlated with age [55]. Similarly, in 376 women with chest pain who ranged in age from 20 to 82 years, larger resting brachial artery diameter, measured by B-mode ultrasonography, was positively associated with age [56]. This relationship was consistent in our sample, where baseline artery diameter was inversely correlated with FMD (ρ = − 0.28, *P* = 0.001) and positively correlated with age (ρ = 0.40, *P* < 0.0001) (data not shown). Thus, to account for these variations in baseline diameter, FMD data were allometrically scaled [57], and results showed similar age and sex values compared to raw FMD percentage change, indicating that differences in age-related changes in baseline vessel diameter do not entirely account for the associations in this sample.

Biological sex was another factor that was predictive of endothelial function; in this study, FMD values were, on average, 1.5% higher in females than males. A study on 457 healthy adults aged 20–91 demonstrated reduced endothelial function with aging and showed that cardiovascular risk occurs 20 years earlier in men [58]. The sex hormone estrogen can exert cardio-protective effects. Estrogen, but not testosterone binding to its receptor, upregulates the release of endothelial NO via increased activity of eNOS in human umbilical vein endothelial cells (HUVEC) [59]. Furthermore, endothelial function declines across the stages of menopause with prolonged estrogen deficiency [60]. Thus, female participants could have had greater cardio-protection, leading to higher FMD in females than males.

Systolic blood pressure was independently associated with FMD. However, in this study, the gradient was nearly zero (B = 0.07), leading to statistical significance but not biological significance. In a group of 5314 Japanese adults (mean age ± SD: 46 ± 13 y, 77.7% male), systolic and diastolic blood pressure were independent predictors of FMD [61], with increases in blood pressure impairing endothelial function. Other classic cardiovascular risk factors, including waist circumference, visceral adipose tissue, and LDL cholesterol, were significant in the correlation analysis but not in the multivariable regression analysis. Our cohort could have potentially been too young to have been exhibiting any of these CVD risk factors in levels high enough to associate with FMD.

The study’s strengths include measuring well-defined outcomes concerning CVD risk factors, including anthropometry, blood pressure, vascular function, diabetes risk, inflammation, and metabolism, using well-accepted methods in a large, predominantly young and healthy population. The present study has some limitations. The 30–45-year-old age bracket was difficult to recruit, which could have affected our analysis; a wider population span is needed to validate our associations even after statistical adjustment. FMD can be affected by the female menstrual cycle [62], and while it was not controlled for in this study, the first day of female participants’ last menstrual cycles were recorded and showed no associations with FMD. The AGE Reader uses ultraviolet light to measure only the AGEs with fluorescent properties in the skin, so it cannot estimate all AGE accumulation, as not all AGEs fluoresce. However, there is a significant correlation between the assessment of AGE accumulation from skin biopsies and the SAF levels calculated by the AGE Reader [4, 5, 10]. Lastly, approximately 9 subjects had dark pigmented skin (Fitzpatrick Type V and VI), which cannot be measured as reliably by the AGE Reader due to higher melanin being present, which absorbs more emission light and more excitation light [63] which can overall affect the SAF reading.

## Conclusions

For the first time, across a predominantly young and healthy adult cohort, increased AGE accumulation quantified by SAF was associated with lower FMD values independent of traditional CVD risk factors. Older age and male sex were also associated with lower FMD values, corresponding with compromised endothelial function. Measurement of SAF is a non-invasive, easy-to-replicate method that could be used as a complementary marker for endothelial function in healthy individuals prior to the appearance of traditional CVD risk markers.

## Electronic supplementary material

Additional file 1.

## Data Availability

The data sets generated and/or analysed during the current study are available from the corresponding author on reasonable request.

## Abbreviations

ACEIs: Angiotensin-converting enzyme inhibitors
AGEs: Advanced glycation end-products
ARBs: Angiotensin II receptor blockers
AU: Arbitrary unit
BH4: Tetrahydrobiopterin
BMI: Body mass index
BP: Blood pressure
CVD: Cardiovascular disease
ECM: Extracellular matrix
ELISA: Enzyme-linked immunosorbent assay
eNOS: Endothelial nitric oxide synthase
FMD: Flow-mediated dilation
HbA_1c_: Glycated hemoglobin
HDL: High-density lipoprotein cholesterol
HOMA-IR: Homeostatic model assessment for insulin resistance
hsCRP: High-sensitivity C-reactive protein
HUVEC: Human umbilical vein endothelial cells
IMT: Intima-media thickness
IPAQ: The International Physical Activity Questionnaire
IQR: Interquartile range
LDL: Low-density lipoprotein cholesterol
MET: Metabolic equivalent task
NADPH: Nicotinamide adenine dinucleotide phosphate
NF-κB: Nuclear factor kappa B
NO: Nitric oxide
O_2_^−^: Superoxide
PWV: Pulse-wave velocity
RAGE: Receptor for AGEs
SAF: Skin autofluorescence
sRAGE: Soluble receptor for advanced glycation end-products
VAT: Visceral adipose tissue
WC: Waist circumference
