# Supplementary material for: Association between endothelial function and skin advanced glycation end-products (AGEs) accumulation in a sample of predominantly young and healthy adults
Source: Cardiovasc Diabetol. 2024 Sep 9;23:332. doi: 10.1186/s12933-024-02428-3 (PMC11386354; doi:10.1186/s12933-024-02428-3)
Supplement: Supplementary file 1 — Additional file 1. [file 12933_2024_2428_MOESM1_ESM.docx]

Table of Contents

[Table S1 Multivariable regression analysis exploring the associations between allometrically scaled FMD and covariates 2](#_Toc165923762)

**Association between endothelial function and skin advanced glycation end-product (AGEs) accumulation in a sample of predominantly young and healthy adults**

| Table S1 Multivariable regression analysis exploring the associations between allometrically scaled FMD and covariates | | | | | | | | | |
| --- | --- | --- | --- | --- | --- | --- | --- | --- | --- |
| **Covariate** | **Unstandardized**  **coefficient** | | **Standardized**  **coefficient** | **t-value** | **P-value** | **95% CI**  **Lower, Upper** | **Obs (N)** | **R^2^** | **Adjusted**  **R^2^** |
|  | **B** | **Std. Error** | **Beta** |  |  |  |  |  |  |
| **Model 1** | | | | | | | | | |
| Intercept | 9.14 | 0.31 | 0.00 | 29.91 | <0.001* | 8.53, 9.74 | 125 | 0.379 | 0.369 |
| Age | -0.05 | 0.01 | -0.46 | -6.37 | <0.001* | -0.06, -0.03 |  |  |  |
| Sex | 1.05 | 0.21 | 0.36 | 4.98 | <0.001* | 0.63, 1.46 |  |  |  |
| **Model 2** | | | | | | | | | |
| Intercept | 5.15 | 2.82 | 0.000 | 1.83 | 0.070 | -0.43, 10.74 | 113 | 0.450 | 0.396 |
| Age | -0.04 | 0.01 | -0.41 | -3.32 | 0.001* | -0.07, -0.02 |  |  |  |
| Sex | 1.21 | 0.26 | 0.42 | 4.69 | <0.001* | 0.70, 1.72 |  |  |  |
| SAF | -0.84 | 0.35 | -0.28 | -2.38 | 0.019* | -1.54, -0.14 |  |  |  |
| WC | 0.002 | 0.01 | 0.02 | 0.18 | 0.860 | -0.02, 0.03 |  |  |  |
| VAT | 0.02 | 0.12 | 0.02 | 0.15 | 0.879 | -0.23, 0.27 |  |  |  |
| Systolic BP | 0.03 | 0.01 | 0.26 | 2.40 | 0.018* | 0.01, 0.06 |  |  |  |
| Heart rate | 0.01 | 0.01 | 0.04 | 0.53 | 0.595 | -0.02, 0.03 |  |  |  |
| Glucose | 0.18 | 0.34 | 0.05 | 0.52 | 0.603 | -0.50, 0.86 |  |  |  |
| LDL Cholesterol | -0.10 | 0.06 | -0.13 | -1.55 | 0.124 | -0.23, 0.03 |  |  |  |
| HbA_1c_ (%) | 0.11 | 0.44 | 0.02 | 0.24 | 0.810 | -0.77, 0.98 |  |  |  |
| Linear multivariable regression analysis was performed to determine the predictors of FMD. Abbreviations: BP, blood pressure; FMD, flow-mediated dilation; HbA_1c_, glycated hemoglobin; LDL, low-density lipoprotein; N, sample size; Obs, observations; SAF, skin autofluorescence; std error, standard error; VAT, visceral adipose tissue; WC, waist circumference. | | | | | | | | | |
